# Complete chloroplast genome of *Trophis caucana* and its phylogenetic implication in Rosales

**DOI:** 10.1080/23802359.2020.1715304

**Published:** 2020-01-21

**Authors:** Pan Deng, Nianyi Sun, Luxian Liu

**Affiliations:** Key Laboratory of Plant Stress Biology, School of Life Sciences, Henan University, Kaifeng, China

**Keywords:** *Trophis caucana*, chloroplast genome, phylogeny inference

## Abstract

*Trophis caucana*, which belongs to Moraceae, is a tree species lived in a humid climate at low and middle altitudes. The complete chloroplast (cp) genome of *T. caucana* was sequenced and assembled in this study. The cp genome is 161,445 bp in length with comprising two copies of inverted region (IR, 25,894 bp) separated by the large single copy (LSC, 89,633 bp) and small single copy (SSC, 20,024 bp) regions. It encodes 111 unique genes, consisting of 77 protein-coding genes, 30 tRNA genes, and four rRNA genes, with 19 duplicated genes in the IR regions. Phylogenetic analysis indicates that *T. caucana* is sister to *Antiaris toxicaria* in Moraceae family.

*Trophis* P. Browne is a small genus in the plant family of Moraceae which includes about nine species with six distributed in Neotropical and three are Palaeotropical. The trees of this genus have alternate leaves, small dioecious green flowers in usually spicate or racemose clusters, and a nearly round thin-fleshed fruit with a single rather large seed (Berg 1988). Previous studies have proved that the phylogeny of this genus was especially problematic, and the species within the genus was polyphyletic in phylogenetic tree based on *ndh*F gene (Datwyler and Weiblen 2004). In the present study, the complete chloroplast genome of *T. caucana* was first sequenced using genome skimming data. The genome sequence was registered into GenBank with the accession number MN868948.

Total genomic DNA was extracted from silica-dried leaves of one *T. caucana* plant collected in Abel Iturralde (Bolivia; 67°58′15.03″W, 14°20′57.10″S) using Plant DNAzol Reagent (LifeFeng, Shanghai) according to the manufacturer’s protocol. A voucher specimen (*Fuentes Claros and Alfredo Fernando 5323*) was deposited at Missouri Botanical Garden Herbarium. High quality DNA was sheared and the paired-end library (≤800 bp) was sequenced on an Illumina HiSeq X10 at Beijing Genomics Institute (BGI, Wuhan, China). The raw data were screened by quality with Phred score <30 and assembled into contigs using the CLC Genomic Workbench (CLC Inc. Aarhus, Denmark). The complete cp genome of *T. caucana* was reconstructed with *Cannabis sativa* (GenBank accession number: KP274871) as a reference and annotated using the software Geneious R11 (Biomatters, Auckland, New Zealand) following description in Liu et al. (2017, 2018). We drew the circular cp genome map of *T. caucana* using the OGDRWA program (Lohse et al. 2013). Phylogenetic tree for 24 whole cp genome sequences of Rosales was constructed using maximum-likelihood (ML) method implemented in RAxML-HPC v8.1.11 on the CIPRES cluster (Miller et al. 2010) with *Lagenaria sceraria* and *Morella rubra* as outgroups.

The cp genome of *T. caucana* was 161,445 bp in length and had a typical quadripartite structure consisting of an 89,633 bp large single copy region (LSC), a 20,024 bp small copy region (SSC) and two 25,894 bp inverted repeats (IRs). Within the genome of *T. caucana*, there are 111 unique genes, including 77 protein-coding genes, 30 tRNA genes, and four rRNA genes, additionally with 19 duplicated genes in the IR regions. Among the 111 genes, six tRNA genes and eight protein-coding genes contain a single intron, and three genes (*rps*12, *clp*P, and *ycf*3) contain two introns. The overall GC content of the total length, LSC, SSC, and IR regions is 35.7%, 33.3%, 28.8%, and 42.6%, respectively. The phylogeny revealed that the five representative genera of Moraceae, including *Antiaris*, *Trophis*, *Ficus*, *Broussonetia*, and *Morus*, formed a highly supported clade, and *T. caucana* is sister to *A. toxicaria* within this family (Figure 1).

**Figure 1.**
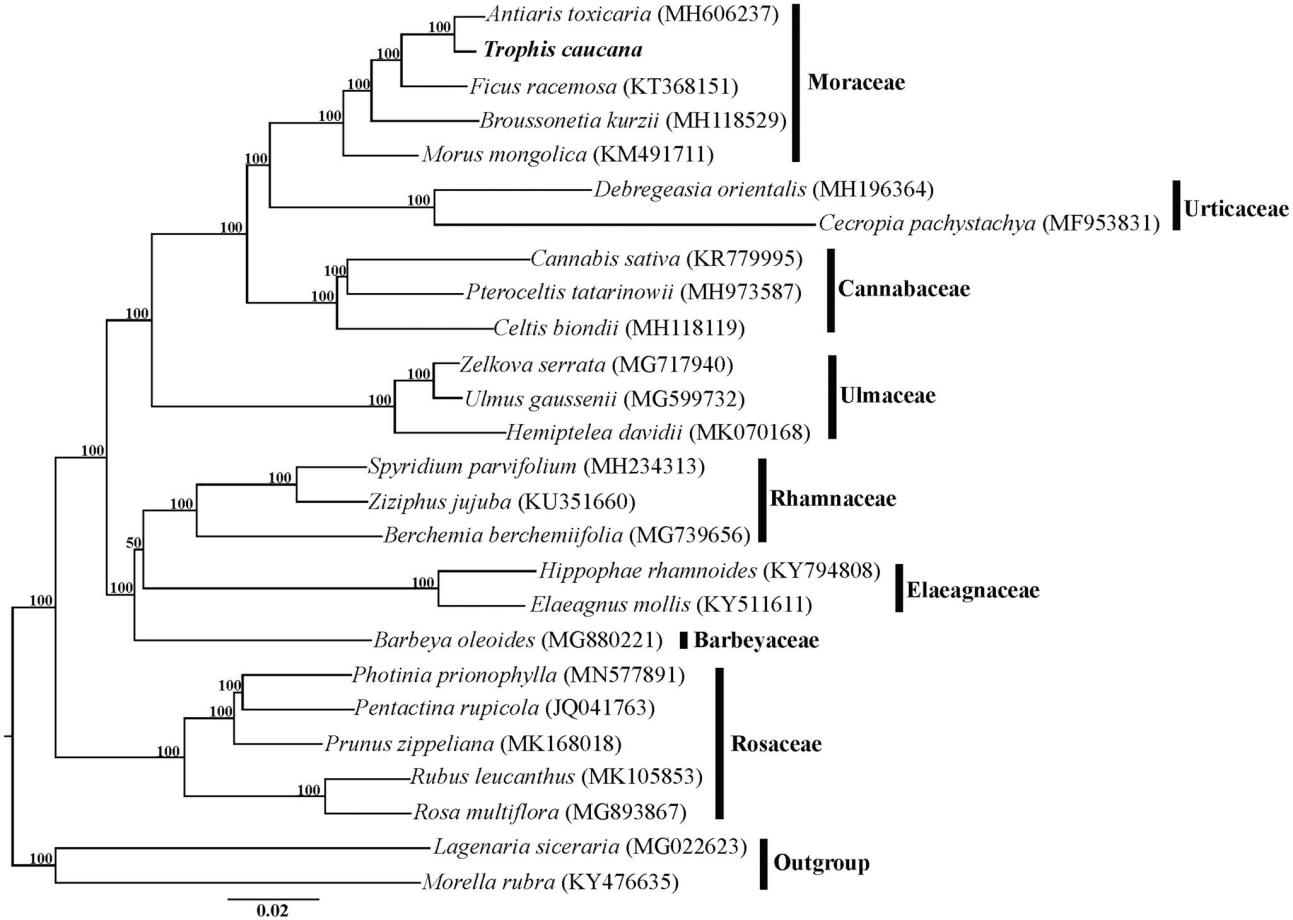
Phylogenetic relationships of Rosales inferred based on whole chloroplast genome sequences. Numbers above the branches represent bootstrap values from maximum-likelihood analyses.

In conclusion, the complete cp genome of *T. caucana* is reported for the first time in this study. It will provide essential and important genetic resources for future better investigation of phylogeny within the genus *Trophis*.
